# Supplementary material for: Breast tumor microbiome regulates anti-tumor immunity and T cell-associated metabolites
Source: bioRxiv. 2024 Nov 2:2024.10.29.620864. Preprint. [Version 1] doi: 10.1101/2024.10.29.620864 (PMC11565759; doi:10.1101/2024.10.29.620864)
Supplement: Supplement 1 [file media-1.pdf]

Supplementary information

Breast tumor microbiome regulates anti-tumor immunity and T cell-associated metabolites

Supplementary figures and legends 1 to 10

Supplementary tables 1 and 2

Supplementary figure 1

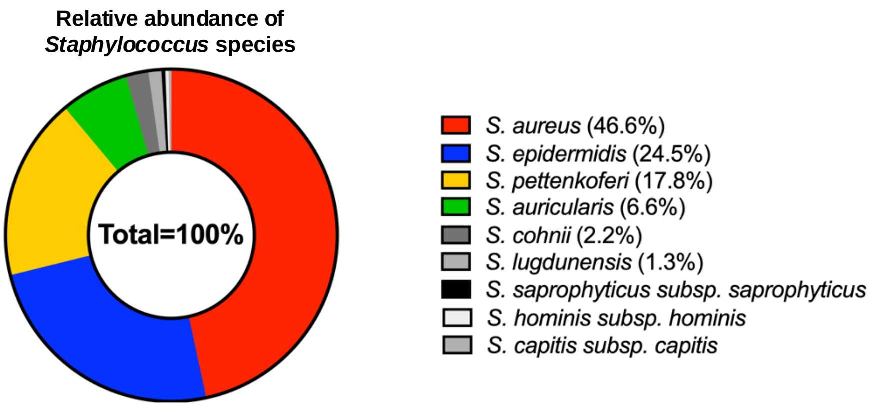

Supplementary figure 1 | The relative abundance of *Staphylococcus* species in human breast tumors. The percentage of *Staphylococcus* species in human breast tumors based on the reads identified in 16S rRNA gene sequencing.

Supplementary figure 2

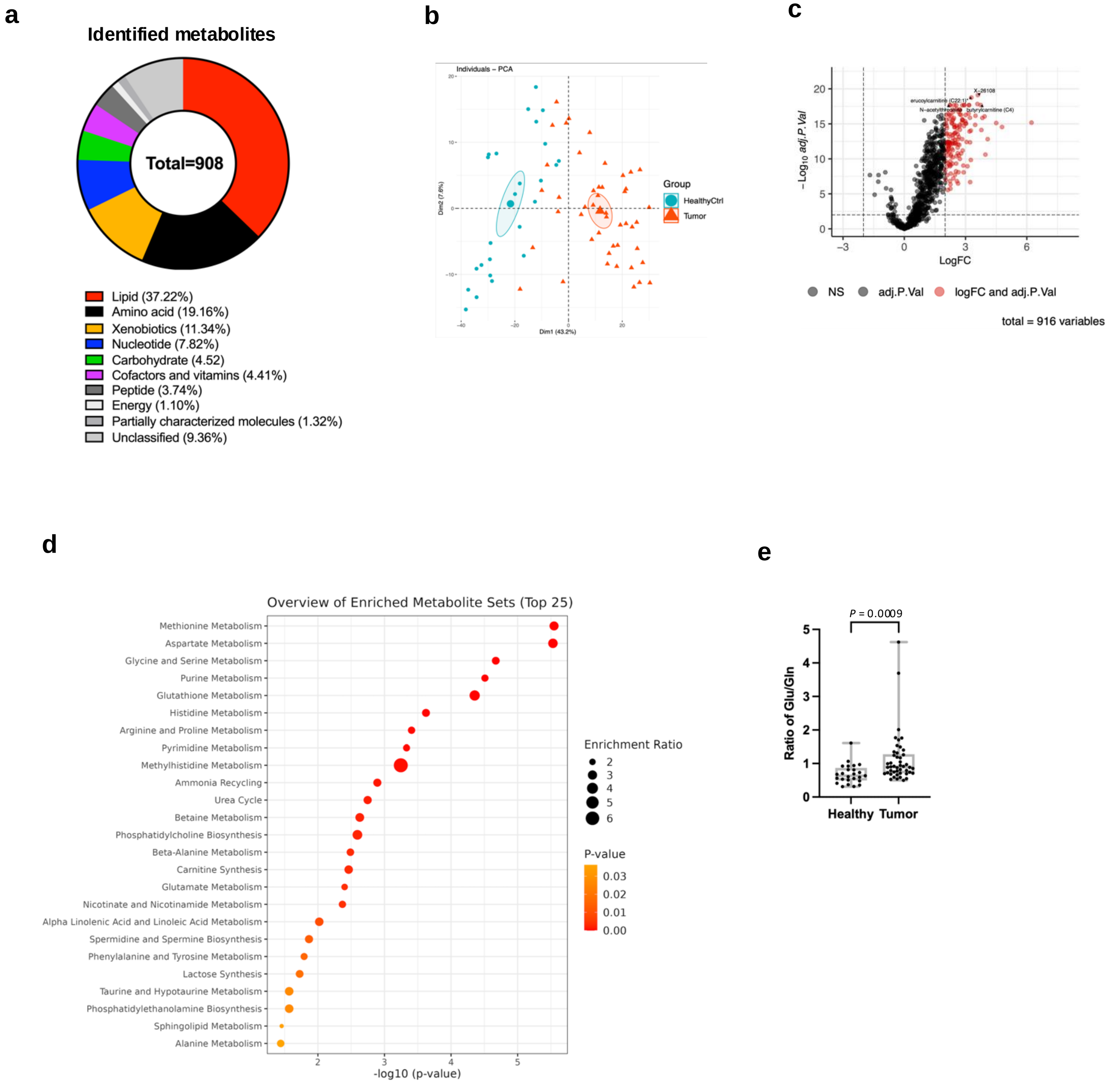

Supplementary figure 2 | Metabolic differences between breast tumors and non-malignant breast tissues. a Composition of the metabolites identified by untargeted metabolomics in human breast tumors and healthy breast tissues. **b** Principal component analysis (PCA) of metabolites in human breast tumors (n=46, in orange) and healthy breast tissues (n=25, in blue). **c** The volcano plot showing the differentially abundant metabolites between breast tumors and healthy breast tissues. Metabolites with a log2 fold change > 2 and –Log10 adjusted p-value > 1.3 are highlighted in red. **d** Top metabolic pathways that are significantly altered in breast tumors compared to healthy breast tissues. **e** Distinct ratios of glutamate (Glu) to glutamine (Gln) between breast tumors and healthy breast tissues.

Supplementary figure 3

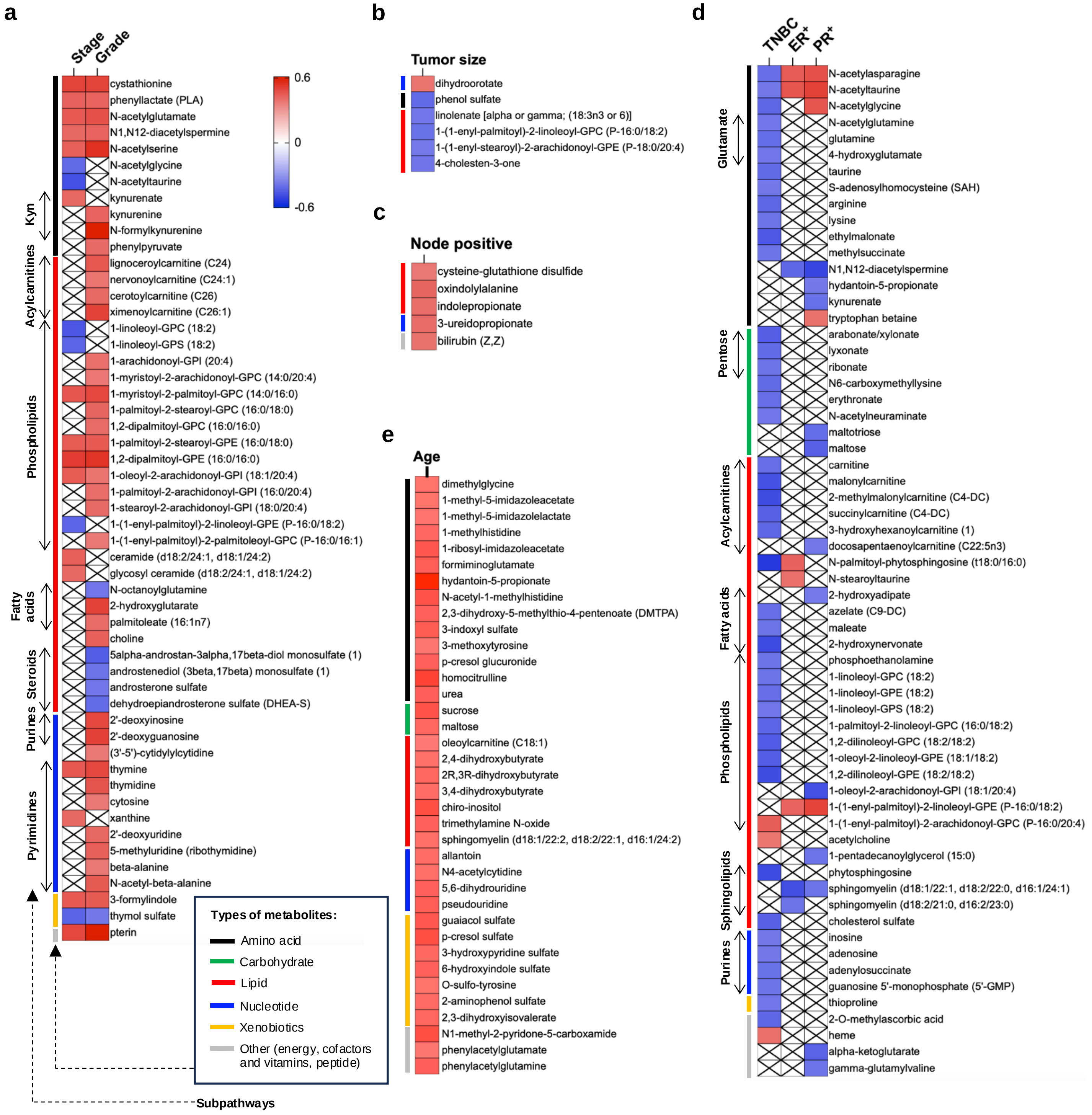

Supplementary figure 3 | Associations between metabolites and clinicopathological features of breast cancer. a-e Heatmaps showing the metabolites significantly associated with cancer stage or histological grade (**a**), tumor size (**b**), lymph node-positive status (**c**), histologic subtypes (**d**), and patient age (**e**) with the color gradient representing correlation. Types and subpathways of metabolites are indicated on the left side of each heatmap.

Supplementary figure 4

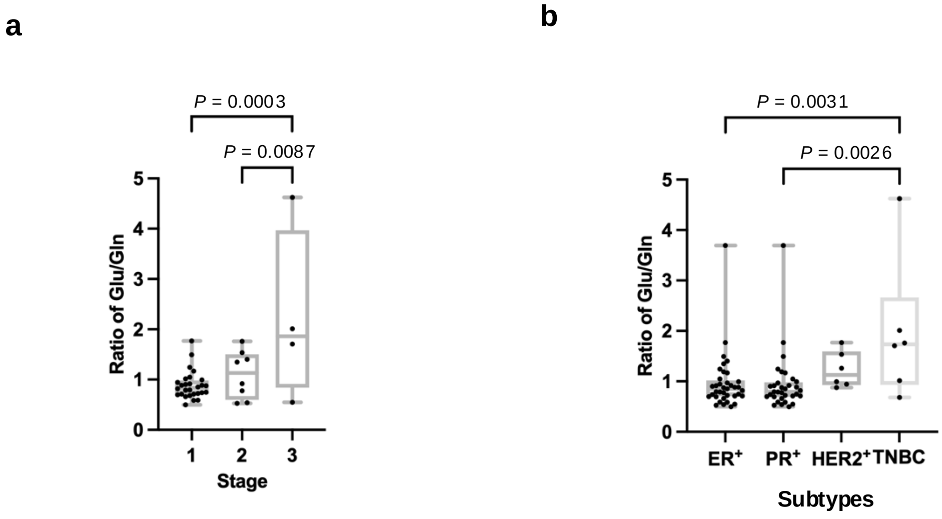

Supplementary figure 4 | The ratio of glutamate to glutamine differs between BC stages and subtypes. a, b Ratio of glutamate (Glu) to glutamine (Gln) in breast tumors across different stages (**a**) and subtypes (**b**). One-way analysis of variance (ANOVA) with multiple comparisons. Only the significant differences are indicated with p-values.

Supplementary figure 5

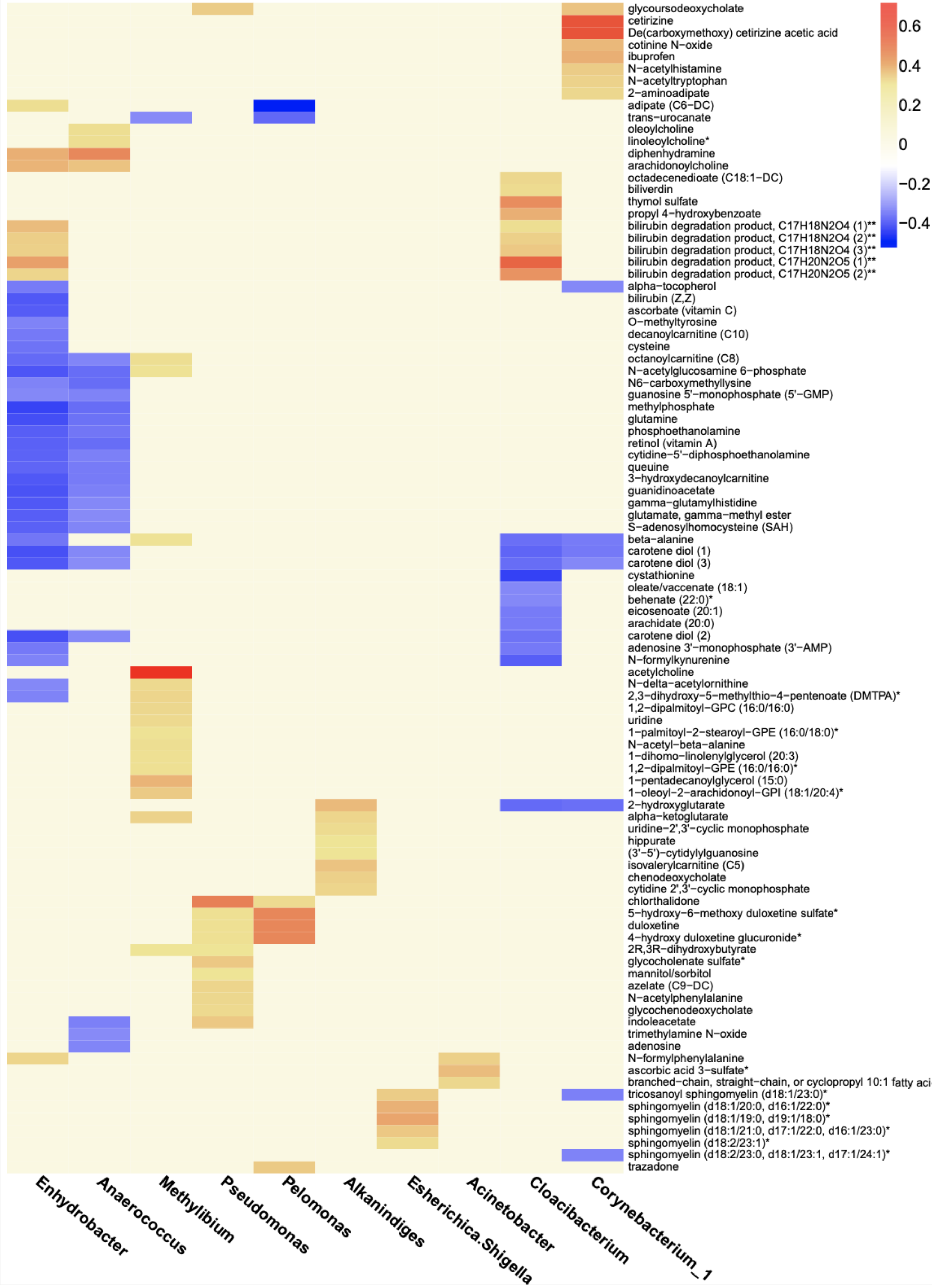

Supplementary figure 5 | Correlations between tumoral bacteria and metabolites. Heatmap displaying significant correlations between the bacterial genera and metabolites within human breast tumors identified by sparse canonical correlation analysis (CCA). The color gradient representing the correlation.

Supplementary figure 6

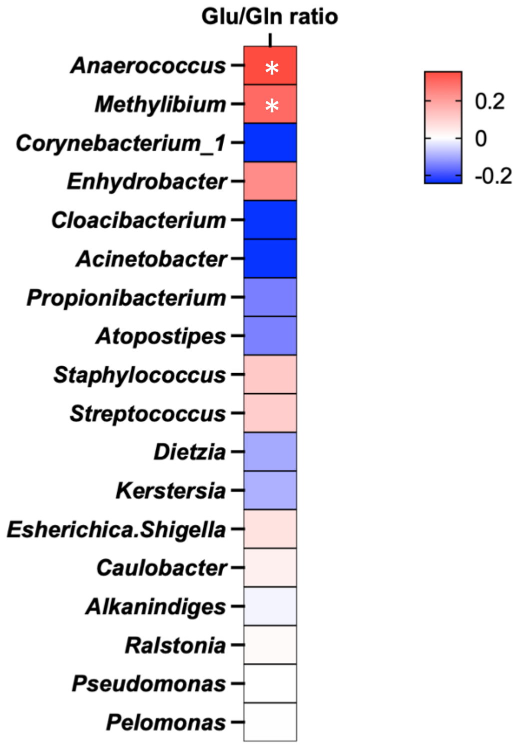

*

Supplementary figure 6 | Correlations between tumoral bacteria and the ratio of glutamate to glutamine. Heatmap illustrating the correlations between bacterial genera and the ratio of glutamate (Glu) to glutamine (Gln). Spearman's rank correlation. *$P < 0.05$.

Supplementary figure 7

a

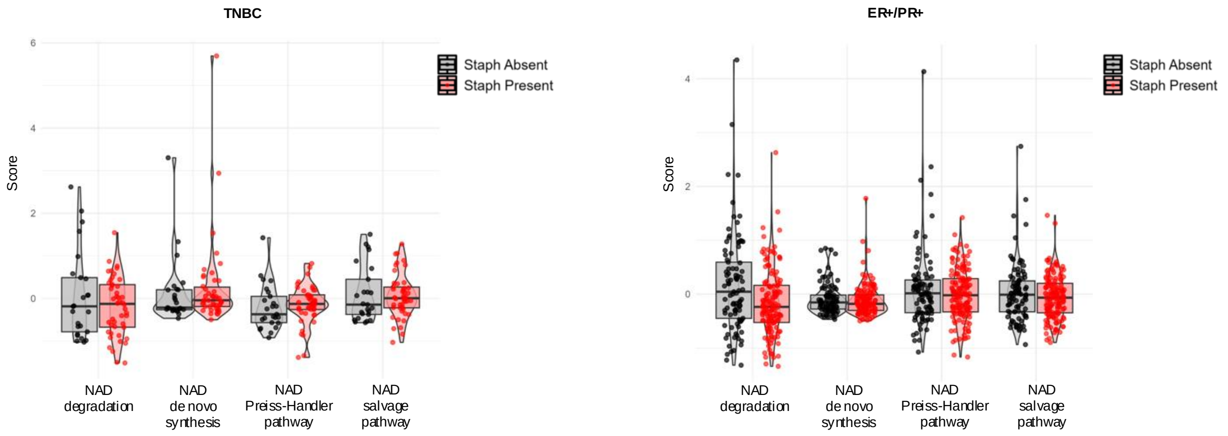

b

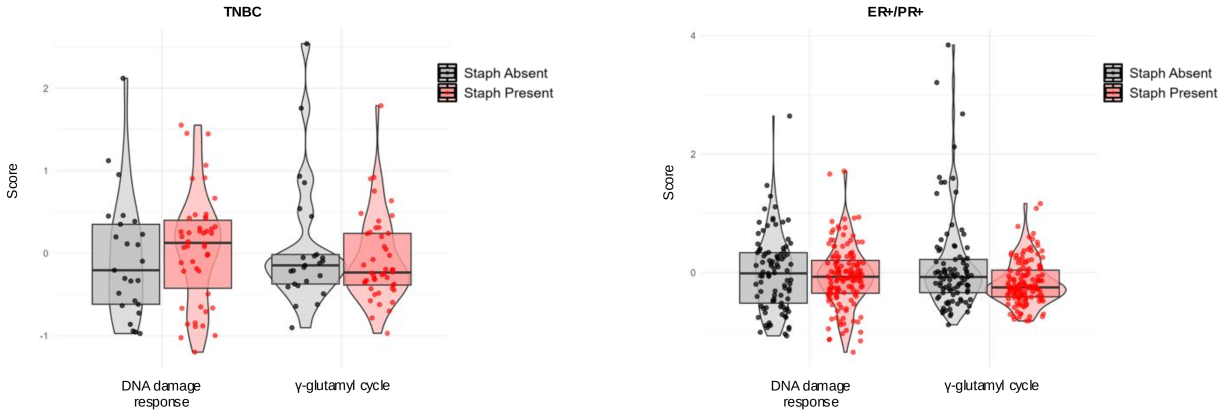

Supplementary figure 7 | Associations between *Staphylococcus* and metabolic pathways in breast cancer. a, b Comparisons of the activity of NAD-related pathways (**a**), DNA damage response (**b**), and γ-glutamyl cycle pathways (**b**) in TNBC (left panel) and ER+/PR+ subtype (right panel) with and without *Staphylococcus* (Staph, denoted in red and black dots, respectively). Z-score transformed signature scores were compared by t-test. All p-values were > 0.05.

Supplementary figure 8

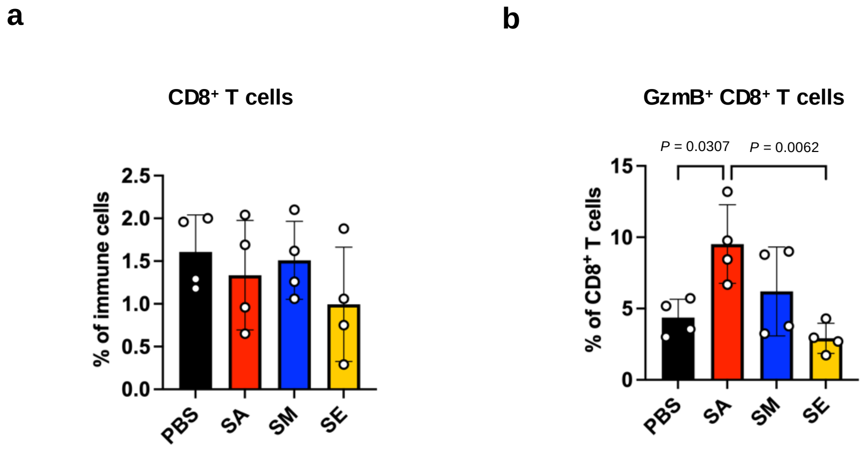

Supplementary figure 8 | Tumoral colonization by *S. aureus* activates CD8+ T cells in 4T1 tumors. a, b 4T1 tumors were colonized by *S. aureus* (SA), *S. mitis* (SM), or *S. epidermidis* (SE), with PBS treatment as a control. Flow cytometry was performed to determine the percentage of $CD8^+$ T cells among immune cells (**a**) and $GzmB^+$ $CD8^+$ T cells among $CD8^+$ T cells (**b**). Only significant differences are indicated with *p*-values. One-way analysis of variance (ANOVA) with multiple comparisons.

Supplementary figure 9

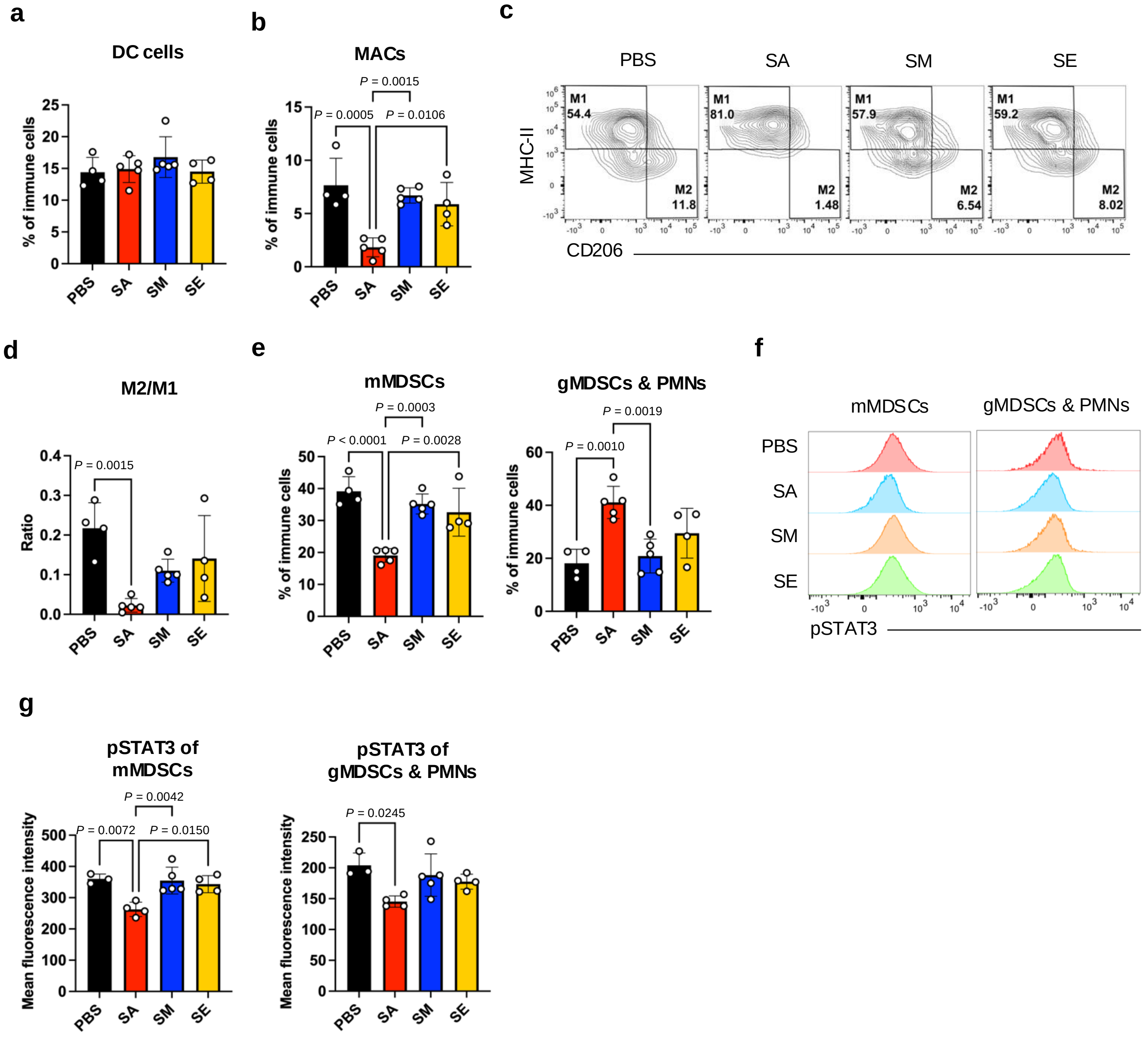

Supplementary figure 9 | Tumoral colonization by *S. aureus* affects innate immune cells in EO771 tumors. a, b, d, e, g Flow cytometry analysis of innate immune cells in the EO771 tumors colonized by *S. aureus* (SA), *S. mitis* (SM), or *S. epidermidis* (SE), with PBS treatment as a control. The analyses including the percentage of dendritic cells (DCs) among immune cells (**a**), the percentage of macrophages (MASc) among immune cells (**b**), the ratio of M2-like to M1-like MACs (**d**), the percentage of monocytic myeloid-derived suppressor cells (mMDSCs) and granulocytic MDSC (gMDSCs)/polymorphonuclear neutrophils (PMNs) among immune cells (**e**), and the mean fluorescence intensity of phosphorylated STAT3 in mMDSCs and gMDSCs/PMNs (**g**). **c**, Representative contour plots showing the gating of M2-like (MHC-II^{low}, CD206^{+}) and M1-like (MHC-II^{high}, CD206^{-}) MACs in the tumors. **f** Representative histograms showing the levels of phosphorylated STAT3 in mMDSCs and gMDSCs/PMNs. One-way analysis of variance (ANOVA) with multiple comparisons (**a**, **b**, **d**, **e**, **g**). Only the significant differences were denoted with *p*-values.

Supplementary figure 10

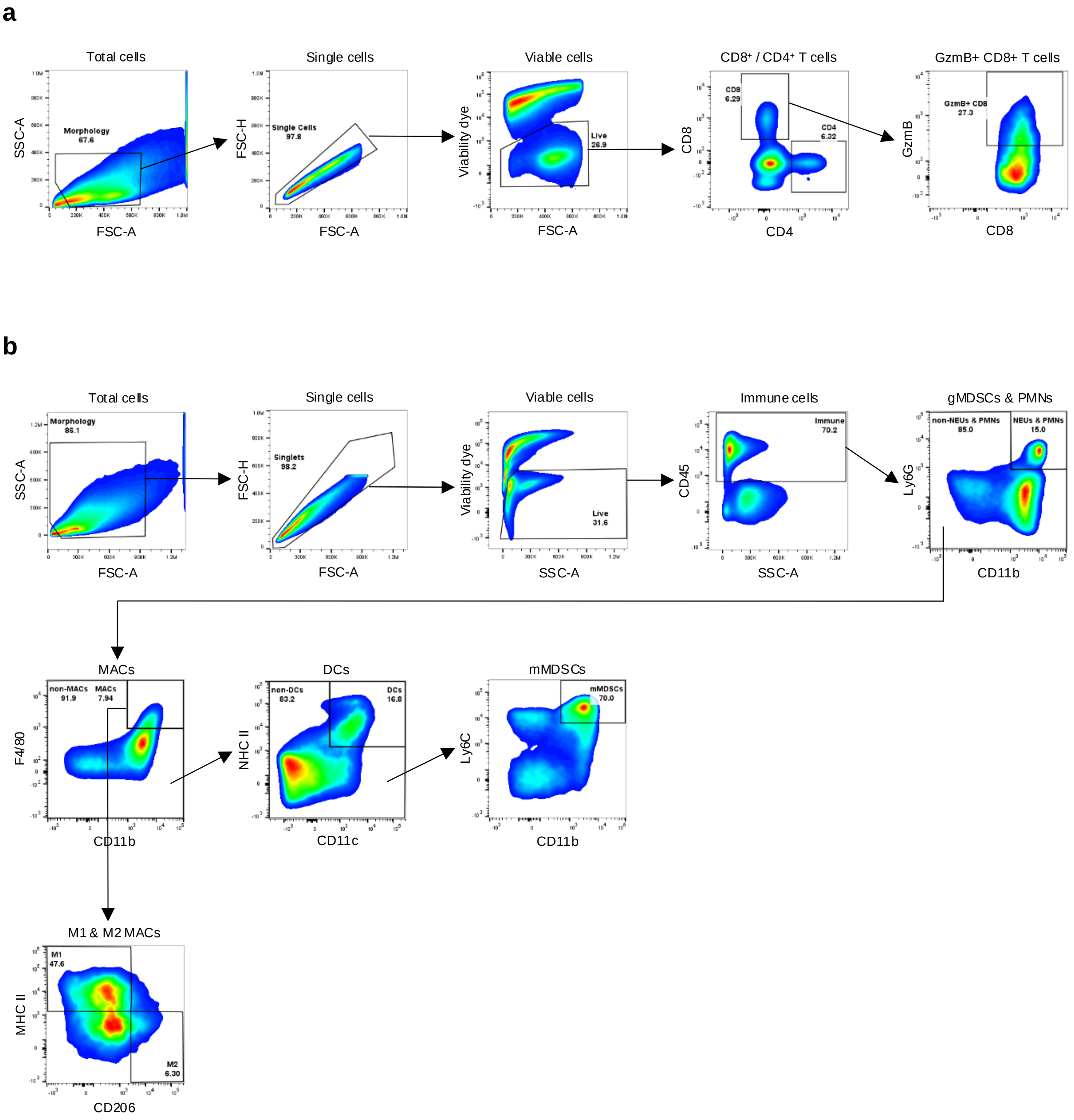

Supplementary figure 10 | Gating strategy for flow cytometry used throughout the manuscript. a, b Gating strategy for GzmB$^+$ CD8$^+$ T cells (**a**) and granulocytic myeloid-derived suppressor cells (gMDSCs) and polymorphonuclear leukocytes (PMNs), macrophages (MACs), M1- and M2-like MACs, dendritic cells (DCs), and monocytic MDSCs (mMDSCs) (**b**).

Supplementary Table 1 | The top 25 metabolites significantly enriched in breast tumors compared to healthy breast tissues.

Metabolites enriched in control breast tissues	Log2 fold change (tumor/control)	Adjusted p-value
erucoylcarnitine (C22:1)*	3.28	1.75E-19
arachidoylcarnitine (C20)*	3.58	1.45E-18
nervonoylcarnitine (C24:1)*	3.16	2.11E-18
docosadienoylcarnitine (C22:2)*	3.18	2.11E-18
butyrylcarnitine (C4)	3.74	2.11E-18
behenoylcarnitine (C22)*	3.15	3.40E-17
glycerophosphoethanolamine	3.01	5.36E-17
glutamate, gamma-methyl ester	3.49	6.99E-17
5-methylthioadenosine (MTA)	3.93	8.58E-17
eicosenoylcarnitine (C20:1)*	3.22	1.35E-16
N-acetylaspartate (NAA)	3.82	1.45E-16
ascorbate (vitamin C)	6.23	6.55E-16
phytosphingosine	4.50	7.50E-16
N-acetylputrescine	3.00	8.86E-16
N-palmitoyl-phytosphingosine (t18:0/16:0)	3.47	1.39E-15
alpha-tocopherol	4.79	2.80E-15
cytidine 5'-diphosphocholine	3.78	7.57E-15
quinolinate	3.26	9.18E-15
ethylmalonate	3.19	1.23E-14
UDP-N-acetylglucosamine/galactosamine	4.08	1.52E-13
N-acetyl-aspartyl-glutamate (NAAG)	3.05	3.85E-13
cystathionine	3.97	2.43E-11
guanosine 5'-monophosphate (5'-GMP)	3.26	1.44E-10
uridine 5'-monophosphate (UMP)	3.09	1.15E-09
glutathione, reduced (GSH)	3.23	2.10E-07

Mann-Whitney U test

Supplementary Table 2 | The top 25 metabolites significantly enriched in healthy breast tissues compared to breast tumors.

Metabolites enriched in control breast tissues	Log2 fold change (tumor/control)	Adjusted p-value
sphingomyelin (d18:1/20:1, d18:2/20:0)*	-0.93	8.33E-09
trans-urocanate	-1.68	2.07E-08
linolenate [alpha or gamma; (18:3n3 or 6)]	-0.89	1.64E-06
sphingomyelin (d18:1/18:1, d18:2/18:0)	-0.65	2.71E-06
sphingomyelin (d18:2/24:2)*	-0.65	3.07E-05
3-hydroxy-2-methylpyridine sulfate	-0.63	5.93E-05
caprylate (8:0)	-0.60	0.00011136
bilirubin degradation product, C17H20N2O5 (2)**	-0.70	0.00030644
octadecadienedioate (C18:2-DC)*	-0.79	0.00051615
branched-chain, straight-chain, or cyclopropyl 10:1 fatty acid (1)*	-0.61	0.00087754
triethanolamine	-0.63	0.00105426
o-cresol sulfate	-0.65	0.00128545
13-HODE + 9-HODE	-0.47	0.00345925
1-(1-enyl-palmitoyl)-2-linoleoyl-GPC (P-16:0/18:2)*	-0.58	0.00820577
androstenediol (3beta,17beta) disulfate (1)	-0.79	0.00902972
3-amino-2-piperidone	-0.64	0.00986265
octadecenedioate (C18:1-DC)	-0.54	0.01034876
sphingomyelin (d18:2/18:1)*	-0.52	0.01066854
4-vinylphenol sulfate	-0.79	0.0109589
N-behenoyl-sphingadienine (d18:2/22:0)*	-0.39	0.01771484
12,13-DiHOME	-0.54	0.0190684
5alpha-androstan-3beta,17beta-diol disulfate	-0.66	0.02234858
fructosyllysine	-0.76	0.02345034
chenodeoxycholate	-0.56	0.03225341
2-aminophenol sulfate	-0.48	0.04465686

Mann-Whitney U test
